# Nanocrystalline BaSnO_3_ as an Alternative Gas Sensor Material: Surface Reactivity and High Sensitivity to SO_2_

**DOI:** 10.3390/ma8095311

**Published:** 2015-09-18

**Authors:** Artem Marikutsa, Marina Rumyantseva, Alexander Baranchikov, Alexander Gaskov

**Affiliations:** 1 Chemistry Department, Moscow State University, Leninskie Gory 1-3, Moscow 119991, Russia; E-Mails: roum@inorg.chem.msu.ru (M.R.); gaskov@inorg.chem.msu.ru (A.G.); 2 Kurnakov Institute of General and Inorganic Chemistry, Leninskiy prospect 31, Moscow 119991, Russia; E-Mail: a.baranchikov@yandex.ru

**Keywords:** barium stannate, sulfur dioxide, nanocrystalline tin dioxide, semiconductor gas sensor, gas-solid interaction

## Abstract

Nanocrystalline perovskite-type BaSnO_3_ was obtained via microwave-assisted hydrothermal route followed by annealing at variable temperature. The samples composition and microstructure were characterized. Particle size of 18–23 nm was unaffected by heat treatment at 275–700 °C. Materials DC-conduction was measured at variable temperature and oxygen concentration. Barium stannate exhibited *n*-type semiconductor behavior at 150–450 °C with activation energy being dependent on the materials annealing temperature. Predominant ionosorbed oxygen species types were estimated. They were shown to change from molecular to atomic species on increasing temperature. Comparative test of sensor response to various inorganic target gases was performed using nanocrystalline SnO_2_-based sensors as reference ones. Despite one order of magnitude smaller surface area, BaSnO_3_ displayed higher sensitivity to SO_2_ in comparison with SnO_2_. DRIFT spectroscopy revealed distinct interaction routes of the oxides surfaces with SO_2_. Barium-promoted sulfate formation favoring target molecules oxidation was found responsible for the increased BaSnO_3_ sensitivity to ppm-range concentrations of SO_2_ in air.

## 1. Introduction

Semiconductor metal oxide (SMO_x_) based gas sensors suffer from the lack of selectivity to target gases. Its fundamental reason might be that typically utilized *n*-type wide band gap binary oxides, such as ZnO, WO_3_, In_2_O_3_ and most often SnO_2_ [1], possess a confined variety of active sites on the surface. These include lattice cations and anions, oxygen vacancies, adsorbed oxygen and hydroxyl species [2]. Material science approaches to increase selectivity are aimed at creating specific active sites on the sensor surface. One of them is chemical modification of binary SMO_x_ by catalytic (noble metal clusters) or acid/base (transition metal oxides) additives [3]. An alternate way is to utilize oxides with more complex composition instead of binary ones as a semiconductor matrix [1]. According to the materials design concept of Hosono *et al.* [4], for a complex oxide to behave as an *n*-type semiconductor, it should be comprised of at least one heavy post-transition cation with the electron configuration (*n*-1)*d*^10^*ns*^0^ (*n* ≥ 4). In a metal oxide the valence band maximum is mainly composed of 2*p*^8^-orbitals of oxygen anions, while conduction band minimum—by *ns*^0^-orbitals of metal cations [5]. If the oxide structure includes a network of heavy post-transition cations, e.g., Zn^2+^, Cd^2+^, In^3+^, Sn^4+^, their spatially extended s-orbitals overlap and form widely dispersed conduction band favoring high electron mobility [6]. Barium stannate is an example of such materials that attracted an interest in gas sensor research [7,8,9,10]. The perovskite-type cubic structure of BaSnO_3_ is challenging for sensor material design by cation substitution in Ba or Sn positions. Semiconductor characteristics determined by the frame of {SnO_6_} octahedra were reported to be varied through either *n*-doping: partial substitution of Ba by La [11,12] or substitution of Sn by Sb [13,14,15], or *p*-doping via Sn substitution by Ni, Cr, *etc*. [12,16,17]. On the other hand, cation substitution in a complex oxide could open wide perspectives for the optimization of surface reactivity by modifying its adsorptive, acid/base and RedOx properties.

Barium stannate is a semiconductor with experimental values of indirect band gap *E_g_* = 3.1–3.4 eV [14,15,18] and direct optical band gap *E_g_* = 3.4–3.5 eV [19]. Density functional theory (DFT) calculations describe band structure of BaSnO_3_ with the conduction band minimum composed of antibonding Sn 5*s*-O 2*s* orbitals slightly contributed by Ba 6*s* orbitals. Valence band is mainly consisted of nonbonding O 2*p* orbitals [20,21]. The band gap was found to increase due to partial substitution of Sn by Sb [14]; increasing the calcination temperature of the materials resulted in narrowing the band gap [22]. The synthesis of BaSnO_3_ is complicated by the need of hard reaction conditions for the formation of perovskite structure. Conventionally obtained by solid-state calcination at T > 1200 °C highly crystalline barium stannate was extensively investigated for good dielectric properties with potential use in capacitors [23,24]. With the development of wet-chemistry synthetic routes under mild conditions, e.g., hydrothermal [25,26,27,28] and lyothermal [29], ion exchange [30], coprecipitation [31] or polymerized complex methods [11,32], BaSnO_3_ was found advantageous for other applications. The latter include infrared luminescence [18], photovoltaics [27], thermoelectric materials [11], humidity detection [17,33] and resistive gas sensors. Promising sensor behavior was reported in the detection of CO and NO [7,8], yet it required high operating temperature of 450–650 °C. Preferred sensitivity to ethanol [31], hydrocarbons [34] and liquefied petroleum gas (LPG) [9] was observed on the background of H_2_, CO, CH_4_ and benzene.

In this work, the effect of annealing temperature on phase composition, microstructure parameters and electric conduction was evaluated for hydrothermally synthesized nanocrystalline BaSnO_3_. Sensing behavior to a number of target gases was tested in comparison with nanocrystalline SnO_2_-based sensors. An increased SO_2_ sensitivity of BaSnO_3_ that exceeded that of tin dioxide was observed for the first time and its origin was studied by *in situ* diffuse reflectance infrared spectroscopy.

## 2. Results and Discussion

### 2.1. Materials Composition and Microstructure Parameters

**Figure 1:**
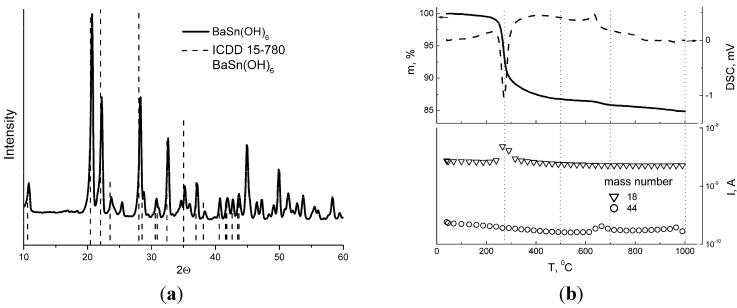
(**a**) XRD pattern of BaSn(OH)_6_ obtained by hydrothermal treatment of barium-tin hydroxide; (**b**) Thermogravimetry (solid line) and differential scanning calorimetry (dashed line) curves for hydrothermally-treated BaSn(OH)_6_ (upper plot) with the results of mass-spectrometry analysis of the outlet gas (lower plot).

The powder yielded from hydrothermal treatment of barium-tin hydroxide consisted of BaSn(OH)_6_ phase with crystallite size *d_XRD_* = 21–27 nm (Figure 1a). The results of as-obtained BaSn(OH)_6_ analysis by thermogravimetry-differential scanning calorimetry with mass-spectrometric detection of outlet gas (TG-DSC-MS) revealed four features (Figure 1b). The largest mass-loss step accompanied by strong endothermic peak at 275 °C is due to water elimination (mass number 18 in Figure 1b) that is consistent with the literature [25]. The amount of desorbed water during the first mass-loss stage, which was completed at ~500 °C, equals to the composition BaSnO_3_·3H_2_O corresponding to BaSn(OH)_6_. At ~700 °C, a small mass-loss (0.5 wt %) along with CO_2_ evolution peak (mass number 44) were observed (Figure 1b). The emergence of an exothermic DSC peak suggests that barium stannate crystallinity increased at this temperature, probably at the expense of some carbonate decomposition. At 1000 °C, additional CO_2_ evolvement with a slight mass-loss were detected by TG-DSC-MS. Thus, annealing BaSn(OH)_6_ at the four featured temperatures was performed to obtain distinct barium stannate samples. Tin dioxide samples synthesized for comparison were processed at the same temperatures.

**Figure 2:**
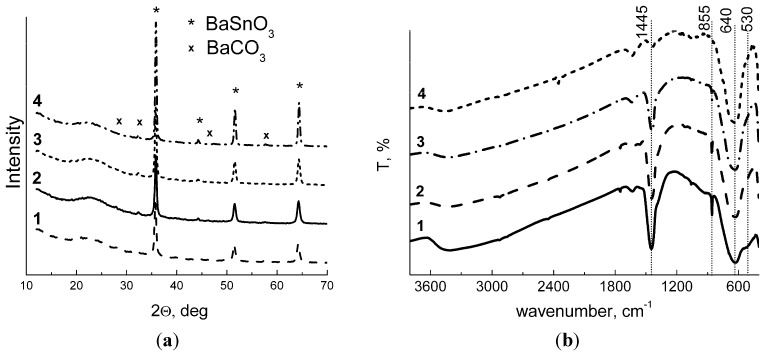
XRD patterns (**a**) and FTIR spectra (**b**) of BaSnO_3_ samples annealed at different temperatures: (1) −275 °C, (2) −500 °C, (3) −700 °C, and (4) −1000 °C.

**Figure 3:**
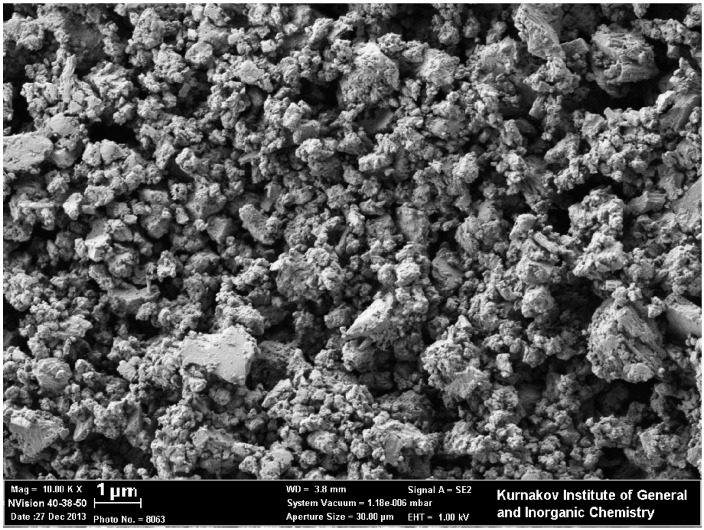
SEM image of BaSnO_3_ sample annealed at 275 °C.

According to X-ray diffraction (XRD) patterns, barium stannate samples consist of well-crystallized cubic perovskite phase with a small impurity of BaCO_3_ (Figure 2a). The appearance of carbonate impurity in BaSnO_3_ was observed elsewhere, despite the hydrothermal synthesis had been performed under inert atmosphere [27]. Fourier-transformed infrared (FTIR) spectra showed the decrease of characteristic carbonate peaks at 1445 cm^−1^ and 855 cm^−1^ with the increase of annealing temperature, so that in the BaSnO_3_-1000 sample carbonate species were hardly detectable (Figure 2b). The major peak at 640 cm^−1^ is due to stretching vibrations of {SnO_6_}-octahedra [26,35]. The intensity of hydroxyl stretching band (3600–3200 cm^−1^) and Sn-OH peak at 510 cm^−1^ diminish with the increase of annealing temperature. As follows from Figure 2a and Table 1, the variation of annealing temperature in the range 275–700 °C had negligible effect on BaSnO_3_ crystallinity and microstructure parameters: the samples have close mean crystallite size (*d_XRD_* = 18–23 nm) and specific surface area of 5–10 m^2^/g estimated by Brunauer-Emmett-Teller (BET) method. Imaging the samples using scanning electron microscopy (SEM) visualized polycrystalline porous structure of the surface (Figure 3). It is likely represented by agglomerates of BaSnO_3_ crystallites, the size of agglomerates widely distributed in the range 0.1–1 μm.

Annealing barium stannate at 1000 °C resulted in a twofold increase of its mean crystallite size and respective decrease of BET area (Table 1). Noteworthy, the improved crystallinity of BaSnO_3_-1000 is coincident with the cleavage of carbonate impurity (Figure 2b). On the other hand, tin dioxide samples were phase-pure (XRD patterns in Supplementary data). Tin dioxide was prepared by the calcination of as-deposited and dried at 50 °C xerogel SnO_2_·nH_2_O, so that its crystallization occurred on the annealing stage. That is why its particle size and surface area were strongly dependent on the annealing temperature value. On the other hand, as-deposited barium-tin hydroxide was hydrothermally treated at 200 °C (pressure was 16–24 bar), that was necessary for BaSn(OH)_6_ phase formation and its further transformation in BaSnO_3_ phase during calcination. It is likely that the well-crystallized character of BaSnO_3_ was determined on the stage of hydrothermal treatment, so that variation of annealing temperature influenced slightly its particle size and surface area. An important factor here seems to be the presence of BaCO_3_ impurity which could be segregated on BaSnO_3_ particles and protect them from thermally induced aggregation. Alternately, the carbonate impurity because of more ionic character and different structure could inhibit ionic diffusion in BaSnO_3_ structure, thus preventing its crystallites growth. This could also explain the coincidence of carbonate disappearance and sharp increase of BaSnO_3_ crystallinity in the material annealed at 1000 °C and their microstructure parameters were strongly dependent on annealing temperature in the whole range (Table 1). Such a contrast in the trends of BaSnO_3_ and SnO_2_ microstructural parameters with annealing temperature could be attributed to different synthetic procedures.

**Table 1:** Samples designation, phase composition and microstructure parameters.

Sample	Annealing T, °C	Crystalline phase ^a^	*d_XRD_* ^b^, nm	BET area, m^2^/g
BaSnO_3_-275	275	BaSnO_3_ (impurity BaCO_3_)	17–19	5–7
BaSnO_3_-500	500		20–22	7–8
BaSnO_3_-700	700		19–23	6–8
BaSnO_3_-1000	1000	BaSnO_3_	40–43	<2
SnO_2_-300	300	SnO_2_	3–6	95–100
SnO_2_-500	500		10–12	20–25
SnO_2_-700	700		16–20	7–10
SnO_2_-1000	700		26–33	<5

Notes: ^a^ from XRD analysis, ^b^ from strongest XRD peaks broadening of the main phase.

### 2.2. Electronic Conductance of BaSnO_3_ in Relation to Temperature and Oxygen Concentration

**Figure 4:**
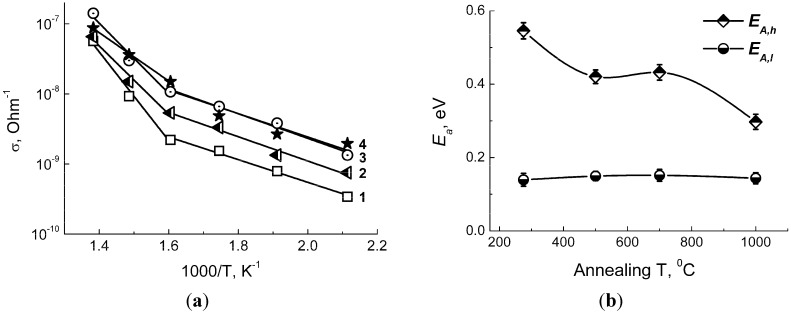
(**a**) Temperature dependence of DC-conductance (σ) of BaSnO_3_ samples annealed at different temperatures: (1) −275 °C, (2) −500 °C, (3) −700 °C, and (4) −1000 °C. (**b**) Activation energy for conductance in relation with annealing temperature of BaSnO_3_. *E_A,h_* is activation energy at higher-temperature (350–450 °C) and *E_A,l_* is activation at lower-temperature (150–300 °C) intervals.

Figure 3a shows the plot of DC-conductance (σ) *vs.* reciprocal temperature for BaSnO_3_-based sensors measured under the atmosphere of purified air. Two regions of conductance with different activation energy values could be outlined from the linear lgσ-1/T dependences (Figure 4a): the lower-temperature one at 150–300 °C and the higher-temperature one at 350–450 °C. Such an effect at close threshold temperature but with different activation energy values was observed in other studies by DC-conduction as well as impedance measurements [16,22]. The lower-temperature activation energy has low value of *E_A,l_* = 0.14–0.15 eV and is independent on annealing temperature of the samples (Figure 4b). According to literature, it corresponds to the conduction via electron hoping by tin cations [16]. The higher-temperature activation energy (*E_A,h_*) tends to decrease in the range ~0.5–0.3 eV with the increase of annealing temperature of BaSnO_3_ from 275 °C to 1000 °C (Figure 4b). Its average value is close to the reported ionization level of double-charged oxygen (V_O_^••^) vacancies (0.37 eV below Fermi level) in barium stannate [19]. The decrease of *E_A,h_* values with the increase of annealing temperature might result from the improved crystallinity that inhibits the formation of deep defect-state levels.

**Figure 5:**
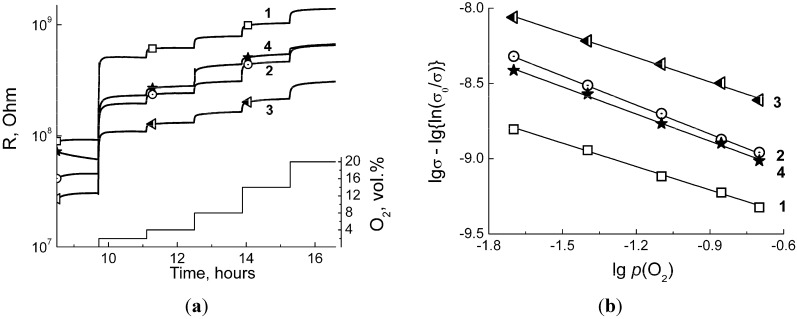

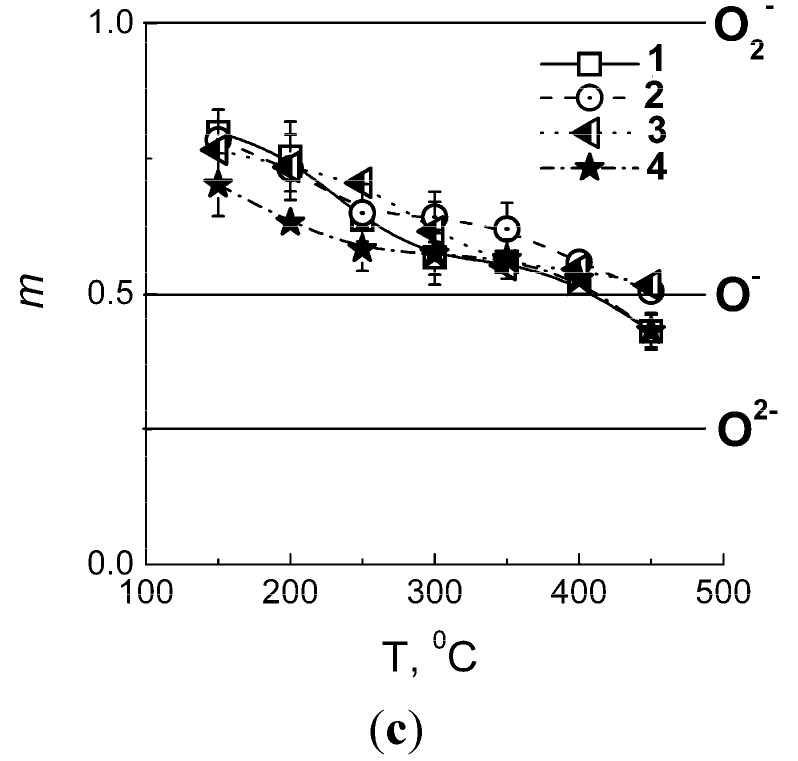
Resistance response to increasing O_2_ concentration measured at 300 °C (**a**); logarithmic conductance *vs.* oxygen partial pressure plot in coordinates of Equation (4) (**b**); and coefficient *m* (Equation (4)) attributed to the type of ionosorbed oxygen species as a function of operating temperature (**c**) for BaSnO_3_ samples annealed at different temperatures: (1) −275 °C, (2) −500 °C, (3) −700 °C, and (4) −1000 °C.

By the resistance measurements at variable oxygen concentration, it was verified that at temperature 150–450 °C barium stannate exhibited *n*-type sensor response. It was evidenced by resistance increase on raising the concentration of O_2_ (oxidative gas) in Ar:air mixture (Figure 5a). At lower temperatures, the samples resistance exceeded the limit of measurements of the electrometer and could not be registered. From the conductance dependence on O_2_ partial pressure the predominant type of ionosorbed oxygen was estimated. To perform it, the data were treated using the ionosorption model developed in [36,37]. According to this, the ionosorption can be considered as the gas molecule interaction with charge carriers at the semiconductor surface:

(1) $$\frac{\beta }{2}O_{2,gas}+\alpha e^{-}=O_{\beta ,surf}^{\alpha -}$$

In a stationary state when the conductance is stabilized, its value depends on the concentration of electrons able to reach the semiconductor surface (*n_s_*), which is dependent on both O_2_ gas partial pressure *p*(O_2_) and the type of ionosorbed species (*α*, *β* coefficients) [36]:

(2) $$n_{s}^{\alpha }=k_{des}/k_{ads}\cdot \Theta \cdot p{\left(O_{2}\right)}^{-\beta /2}$$

where *k_ads_* and *k_des_* are the rate constants of oxygen ionosorption and desorption, respectively, and Θ is the surface coverage by ionosorbed species. Applying complicated expressions for surface coverage to two model approximations, the conductance should be linearly dependent on oxygen partial pressure in logarithmic coordinates [37]

(3) (i) for small crystallites: lgσ−lg(1−σ/σ0)=const−m⋅lg p(O2)

(4) (ii) for large crystallites: lgσ−12lg{ln(σ/σ0)}=const−m⋅lg p(O2)

where *σ* is conductance in presence of oxygen and *σ_0_* is conductance in Ar in absence of oxygen. The approximation (i) is applied to fully depleted semiconductor particles with radius less than Debye length, while the case (ii) refers to large enough particles with size larger than Debye length and, hence, with the separation between depleted surface and not depleted bulk regions. Here it is assumed that the effect of ionosorption on electron mobility is minor in comparison with that on electron concentration [37]. Parameter *m* = β/2α is relevant to the type of ionosorbed species. For example, on the surface of tin dioxide the ionosorption route is known to be dependent on temperature: (*a*) at 100–170 °C it yields mainly molecular O_2_^−^ species, (*b*) at 200–350 °C—atomic O^−^ and (*c*) at higher temperatures—fully ionized O^2−^ ionosorbates [36].

The logarithmic plots of barium stannate conductance *vs.* oxygen partial pressure could be adequately fitted only using Equation (4), *i.e.* in the approximation to large grains (Figure 5b). This seems reasonable regarding BaSnO_3_ crystallite sizes in Table 1, yet no data were found in literature for the Debye length of this material. In Figure 5c, the calculated slope values (*m*) are summarized with the attribution to the type of ionosorbed oxygen species. As can be seen, within the fitting errors the ionosorption mode was similar for all BaSnO_3_ samples and independent on annealing temperature. It was, however, dependent on the temperature value at which the interaction with gas phase was investigated. The decrease of slope value from *m* = 0.7–0.8 (at 150–200 °C) to *m* ≈ 0.5 (at 300–400 °C) could be interpreted as the transformation of ionosorbed oxygen from mixed O_2_^−^/O^−^ to predominantly atomic O^−^ species following the increase of temperature (Figure 5c). This trend partially coincides with that considered above for tin dioxide. At 450 °C, the slope shifted to *m* ~ 0.4, probably due to appearance of atomic O^2−^ ionosorbates on the surface at high temperature.

### 2.3. Gas Sensitivity of BaSnO_3_ in Comparison with SnO_2_

**Figure 6:**
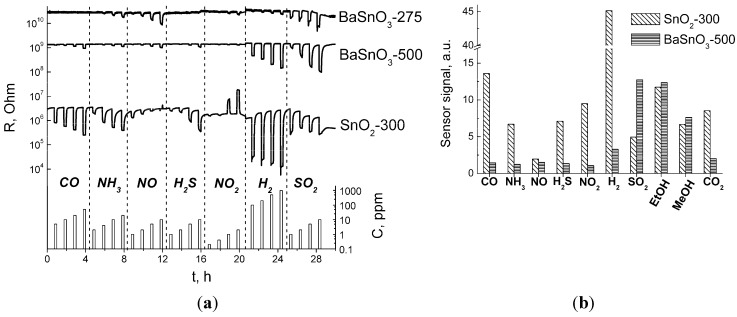
(**a**) Dynamic resistance plot of BaSnO_3_-275, BaSnO_3_-500 and SnO_2_-300 sensors to the increasing concentrations of various target gases in air measured at 300 °C. (**b**) Sensor signals of BaSnO_3_-500 and SnO_2_-300 to target gases in air: CO (50 ppm), NH_3_ (20 ppm), NO (10 ppm), H_2_S (2 ppm), NO_2_ (2 ppm), H_2_ (100 ppm), SO_2_ (10 ppm), EtOH (20 ppm), MeOH (20 ppm) and CO_2_ (1%); T = 300 °C.

Figure 6a shows the dynamic response for BaSnO_3_-based sensors and SnO_2_-300 reference sensor to ppm-range concentrations of various target gases measured at 300 °C. (The data for BaSnO_3_-700 are not shown, since its resistance intersected in some cases with that of SnO_2_-300, which would sophisticate the representation. However, its sensing behavior was quite similar to that of BaSnO_3_-500. The sample BaSnO_3_-1000 was almost insensitive to target gases.) Both materials demonstrated *n*-type response, *i.e.*, resistance decreases in presence of reductive target gases. Taking into account that BaSnO_3_ and SnO_2_ behave as *n*-type semiconductors because of anion vacancies V_O_^••^, similar sensing mechanism could be anticipated for the interaction with reductive gases as it had been done elsewhere [7,38]. In principle, this process includes oxygen ionosorption during exposure in air and subsequent partial reduction of the ionosorbed species when the surface gets in contact with target gas molecules. Yet, barium stannate did not display any resistance growth on exposure to NO_2_ that would have been expected for an *n*-type sensing material (Figure 6a). The contrast with SnO_2_ may be due to smaller charge carrier concentration in BaSnO_3_ and/or lower energy of the donor-state level. Actually, the position of V_O_^••^ defect level at 0.114–0.140 eV below Fermi level was reported for bulk tin dioxide [39], which is less than that in barium stannate (0.37 eV [19]). Taking into account that its work function (>5 eV [40,41]) is also higher in comparison with SnO_2_ (4.8 eV [42]), the deeper position of V_O_^••^ donor level could prevent the ionosorption of oxidizing NO_2_ molecules on barium stannate. Figure 6b compares the sensor signal values of BaSnO_3_-500 and SnO_2_-300 to a fixed concentration of the target gases tested at 300 °C. In general, the gas sensitivity of barium stannate was lower than that of tin dioxide. The sensor signal values of BaSnO_3_ to CO, NO, H_2_ and NO_2_ are well below *S* = 5, which is in agreement with previous works [7,8,9,10,31,38]. The higher sensitivity of tin dioxide observed in most cases must be contributed by its much larger dispersity in comparison with BaSnO_3_ (Table 1). A comparable sensitivity was noted in the detection of alcohols (Figure 6b). The response to NO having close values at 300 °C was distinct at lower temperature: the sensitivity of SnO_2_ increased strongly at lower temperature 100–200 °C (Supplementary data). It was only to SO_2_ that barium stannate demonstrated evidently higher sensitivity than tin dioxide (Figure 6b).

Regarding the selectivity parameter, it is appropriate to compare the sensor signals to close concentrations of target gases. From Figure 6b it can be concluded that unlike SnO_2_, the sensitivity of barium stannate is much more dependent on the chemical nature of the target gas molecule, rather than on its concentration. Among the tested gases BaSnO_3_ was more sensitive to SO_2_ and alcohols (Figure 6b). In contrast to it, tin dioxide displayed highest signals to hydrogen since it had the highest concentration (100 ppm), then to CO (50 ppm) and lower signals to other gases with lower concentrations. The exceptions from such a correlation are the tests to H_2_S, which is highly adsorptive and reactive gas, NO_2_ (since it is an oxidative gas) and CO_2_ (the gas with neither reductive, nor oxidative properties). The increased sensitivity of barium stannate to EtOH in comparison to CO, H_2_, LPG and benzene was reported in [31], which is consistent with the present results. The novel finding in this work is that the sensitivity of BaSnO_3_ to SO_2_ (10 ppm) exceeds its sensitivity to EtOH (20 ppm), despite the latter was tested in a higher concentration. Thus, it can be outlined that barium stannate possessed selectivity to SO_2_ in comparison to other target gases tested, however to confirm it the experiments on the detection of mixtures of target gases would be needed. It should be noted that there is a potential to improve selectivity of barium stannate-based sensors via it surface modification by catalytic (noble metal) clusters or acid/base (transition metal oxides) additives, doping of BaSnO_3_ via cation substitution in Ba or Sn sites that would modify surface reactivity, or by depositing filtering membranes (alumina, silica, *etc.*). For example, it was shown that modification by Pt increases the selectivity to LPG [9], while using Al_2_O_3_ additive the selectivity of barium stannate to benzene was improved [38].

**Figure 7:**
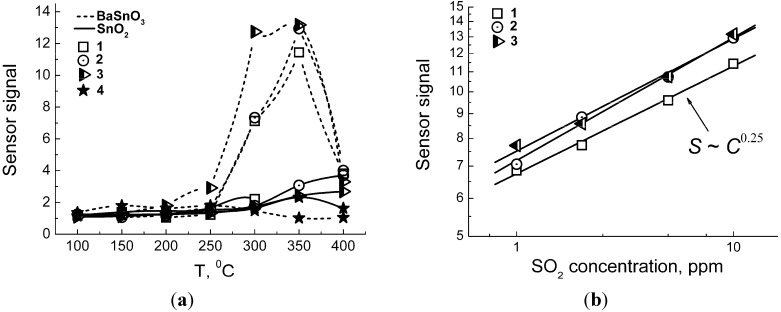
(**a**) Temperature dependence of sensor signals to SO_2_ (10 ppm) for BaSnO_3_ and SnO_2_ samples. (**b**) Logarithmic plot of sensor signals measured at 350 °C *vs*. SO_2_ concentration for BaSnO_3_ samples annealed at different temperatures: (1) −275 °C, (2) −500 °C, (3) −700 °C, and (4) −1000 °C.

In Figure 7a, the temperature plots of sensor signals to SO_2_ are summarized for all materials studied. Barium stannate samples annealed at 275–700 °C displayed close responses with the maximum at 300–350 °C. Some improvement of sensitivity was noted with the increase of BaSnO_3_ annealing temperature to 700 °C. The lack of sensitivity of BaSnO_3_-1000 could be due to its too low surface area. It could be noted that carbonate impurity that inhibited BaSnO_3_ particle size growth likely played a favorable role for the gas sensitivity of samples annealed at 275–700 °C. In this context, it is important that the impurity is inactive it the process of interaction with the target gases, as is discussed below. The most interesting observation was that the responses of BaSnO_3_ annealed at 275–700 °C exceeded in several times those of SnO_2_ samples, despite the latter were favored by up to one order of magnitude larger BET surface area and smaller particle size (Table 1). The sensor signal of BaSnO_3_ follows the exponential dependence on SO_2_ concentration (Figure 7b).

To conclude, the sensitivity of barium stannate to most target gases tested is in general lower than that of tin dioxide. This is most likely because the latter possessed much larger surface area available to interact with the gas phase. Taking into account the simpler aqueous deposition route to obtain SnO_2_ with BET surface area of the order 100 m^2^/g than the hydrothermally-assisted synthesis of barium stannate giving materials with BET surface area up to 10 m^2^/g, BaSnO_3_-based materials have limited perspectives to be applied in gas sensing field and tin dioxide would remain being the most utilized sensing material in the detection of common atmospheric pollutants (CO, H_2_S, NH_3_, hydrocarbons). However, in the detection of SO_2_ traces in air, BaSnO_3_ turns out to be significantly more sensitive and selective than SnO_2_. This advantage can possibly be enhanced by increasing the dispersity of BaSnO_3_ and modifying its surface by catalytic additives of noble metals. Noteworthy, among the materials studied for SO_2_ detection, tin dioxide and, to a lesser extent tungsten oxide, were previously regarded as the most efficient ones, their sensitivity increasing due to surface modification by Ag, Pd and Pt additives [43].

### 2.4. DRIFT Study of the Materials Interaction with SO_2_

**Figure 8:**
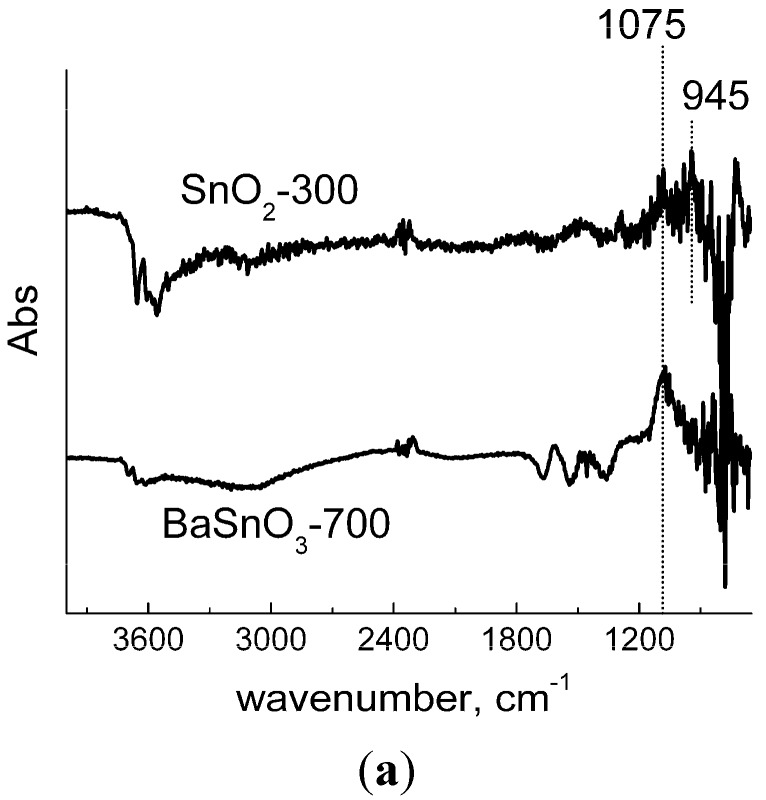

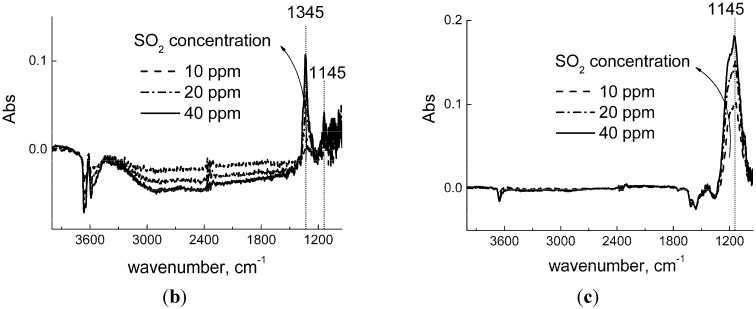
DRIFT spectra of SnO_2_-300 and BaSnO_3_-700 samples exposed to SO_2_ (40 ppm) at room temperature (**a**) DRIFT spectra of SnO_2_-300 sample (**b**) and BaSnO_3_-700 sample (**c**) exposed to increasing concentration of SO_2_ (10–20–40 ppm) at 300 °C.

The reason for increased SO_2_ sensitivity of barium stannate was found from *in situ* study of the materials interaction with the target gas by means of diffuse reflectance Fourier-transform infrared spectroscopy (DRIFT). The interaction was investigated on the example of BaSnO_3_-700 and SnO_2_-300 samples as the most sensitive ones, at room temperature (adsorption regime) and at 300 °C (reaction regime). From the spectra of samples exposed to 40 ppm SO_2_ at room temperature (Figure 8a), it could be inferred that the adsorption proceeds in a not dissimilar manner on both materials. In presence of target gas, the peak arises at 1075 cm^−1^, which is more pronounced for BaSnO_3_. It can be ascribed to chemisorbed SO_2_ [44] as well as to *v*_3_ asymmetric stretching vibrations of S-bound sulfite species [45]. The peak of O-bound sulfite (945 cm^−1^ [45]) was also prominent on the spectrum of SnO_2_ (Figure 8a). Thus, molecular chemisorption (e.g., on cation sites) along with sulfite species formation due to SO_2_ bonding with oxide anions took place on the surface of BaSnO_3_ and SnO_2_ at room temperature. The depletion in OH-stretching vibration region (3600–3200 cm^−1^) could result from surface hydroxyls elimination due to competitive SO_2_ chemisorption.

The gas-solid interaction routes, however, were strongly distinct at raised temperature. On the surface of SnO_2_, molecular SO_2_ adsorption was the predominant interaction route at 300 °C, as deduced from the evolvement of the peaks at 1345 cm^−1^ and 1145 cm^−1^ (Figure 8b). This doublet is characteristic of asymmetric (*v*_3_) and symmetric (*v*_1_) stretching bands, respectively, of adsorbed SO_2_ [46]. The weak adsorption character is indicated by a little shift of the peaks centers to lower wavenumbers in comparison with gas-phase molecule (*v*_3_ = 1360 cm^−1^, *v*_1_ = 1151 cm^−1^ [46]). The alteration of SO_2_ adsorption mode on tin dioxide surface can be explained by weakening the molecule-to-surface binding at raised temperature that prevents strong chemisorption, which was observed at room temperature. On the other hand, desorption of hydroxyl species was as well intensified at raised temperature, as follows from the increased depletion of OH-stretching band at 3600–3400 cm^−1^ (Figure 8b). This could provide more adsorption sites for SO_2_ and account for the increased target gas adsorption in comparison with room-temperature situation, as follows from the comparison of S-O bands intensities at 1350–900 cm^−1^ in Figure 8a,b.

The main feature on the DRIFT spectra of barium stannate interacting with SO_2_ at 300 °C was the evolution of a strong band centered at 1145 cm^−1^ with a shoulder at ~1200 cm^−1^ and a satellite at 1050 cm^−1^ (Figure 8c). In contrast to SnO_2_, the absence of a peak at 1340–1350 cm^−1^ ruled out that molecular adsorption took place on BaSnO_3_ surface. According to [45,47], this wavenumber region corresponds to asymmetric (*v*_3_) stretching vibration band of sulfate ions. The doublet of 1145 cm^−1^ and 1050 cm^−1^ peaks is an indicative of monodentate sulfate, while the shoulder at 1200 cm^−1^ suggests the presence of bidentate species as well [45]. Moreover, the set of the three peaks is typical to bulk BaSO_4_ [48]. Hence, SO_2_ oxidation is the predominant process on the surface of BaSnO_3_ at raised temperature. In comparison with tin dioxide, the presence of Ba^2+^ cations seems to play the key role in determining the interaction route. Bulk BaSO_4_ is known as a stable compound. The formation of its lattice fragments might be the driving force for promoting SO_2_ gas oxidation on the surface of BaSnO_3_. This is believed to be the reason for increased sensitivity of barium stannate to SO_2_ at raised temperature. With respect to oxygen ionosorption type (Figure 5c), the following scheme could be suggested for the improved sensing process at the temperature of maximum sensitivity (300–350 °C):

(5) $$Ba_{lat}^{2+}+O_{lat}^{2-}+O_{surf}^{-}+SO_{2,gas}\to BaSO_{4,surf}+e^{-}$$

Although a weak negative peak of hydroxyl groups was still observed at 3640 cm^−1^, no depletion in carbonate-vibration range (~1450 cm^−1^) was detected (Figure 8c). This indicates that the impurity of BaCO_3_ was not involved in the interaction of barium stannate with SO_2_. Noteworthy, no sulfate-related phases could be found after gas–solid interaction by XRD analysis.

## 3. Experimental Section

Barium stannate was synthesized by aqueous coprecipitation of freshly deposited stannic acid and barium hydroxide followed by hydrothermal treatment [26]. Stannic acid was obtained by dropwise addition of 1 M aqueous ammonia solution to stirred 0.3 M solution of tin (IV) chloride prepared from SnCl_4_·5H_2_O (Sigma-Aldrich, St. Louis, MO, USA) until pH ~ 6 was reached. The deposit of SnO_2_·nH_2_O was washed from chloride-ions using deionized water and centrifugation. Then it was peptized in a minimum volume of 10 M aqueous ammonia (pH > 9) and added to 0.2 M Ba(OH)_2_ solution. The latter was prepared using Ba(OH)_2_·8H_2_O (Sigma-Aldrich, St. Louis, MO, USA) and deionized water after degassing under Ar at 70–80 °C. The mixture of precursors with the molar ratio Ba:Sn = 1:1 was stirred at 60 °C for 1 h under Ar flow as a protecting atmosphere. The obtained heavy white precipitate was placed in a teflone-line autoclave and hydrothermally treated at 200 °C (*p* = 16–24 bar) for 2 h in a microwave-heated reactor Berghof MWS-3+ (Berghof, Eningen, Germany). Next, it was washed from unreacted barium hydroxide until pH ~ 7, decanted and dried in air at 50 °C for 24 h. The resultant BaSn(OH)_6_ powder was divided into four parts, which were annealed in air for 24 h at different temperatures: 275 °C, 500 °C, 700 °C, 1000 °C. The annealing temperature values were chosen on the basis of thermogravimetric analysis.

Nanocrystalline tin dioxide samples were obtained from stannic acid deposited as described above. The washed deposit of SnO_2_·nH_2_O was dried in air at 50 °C. The xerogel was separated into four parts, which were annealed at different temperatures: 300 °C, 500 °C, 700 °C, and 1000 °C.

X-ray diffraction was registered using DRON-3M instrument (Burevestnik, St.-Petersburg, Russia). Crystallite size (*d_XRD_*) was calculated from the broadening of the strongest reflexes using Scherer equation, wavelength *λ* = 1.54051 Å (Cu Kα_1_ radiation). Scanning electron microscopy (SEM) was performed using Carl Zeiss NVision 40 instrument (Carl Zeiss NTS, Oberkochen, Germany) with accelerating voltage 1–30 kV. The specific surface area of powders was estimated by nitrogen adsorption method using the Brunauer-Emmett-Teller (BET) model with the Chemisorb 2750 instrument (Micromeritics, Norcross, GA, USA). Powders (100–150 mg) were tested after pretreatment under He flow at 200 °C, the probe gas was N_2_ (30 vol %):He. Thermogravimetric-differential scanning calorimetric analysis with mass-spectrometric detection of outlet gas (TG-DSC-MS) was performed on the NETZSCH STA 409 PC instrument (NETZSCH, Selb, Germany). Powder (20 mg) in an alumina crucible (6 mm diameter) was heated to 1000 °С with the rate of 10 °С/min under synthetic air flux (10 mL/min).

FTIR analyses were performed using Frontier (Perkin Elmer, Massachusetts, MA, USA) spectrometer in transmittance and diffuse reflectance (DRIFT) modes. The spectrometer works under ambient conditions with automatic H_2_O/CO_2_ compensation (spectrometer self-test every 15 min). In the transmittance mode, the powders (~1 mg) were ground with 50 mg KBr and pressed into pellets; the spectra were registered in 4000−400 cm^−1^ wavenumber region with resolution 4 cm^−1^ and accumulation of 4 scans. In the DRIFT mode, the gas-solid interactions were studied. Powders (30 mg) in an alumina crucible (5 mm diameter) were placed in a heated flow camera HC900 (Pike Technologies, Fitchburg, MA, USA). The camera was mounted in the DiffusIR annex of the spectrometer. Prior to the tests, the samples were pretreated under purified air flux (100 mL/min) for 1.5 h at a constant temperature corresponding to the subsequent measurement. Generator of purified air (model “1,2–3,5”, Himelectronica, Moscow, Russia) was used as the carrier gas source, contaminations level according to the manufacturer guarantee not exceeding: H_2_O—10 ppm, CO_2_—2 ppm, and hydrocarbons—0.1 ppm. The pretreatment completeness was checked by the absence of spectral changes due to water desorption during 15–30 min. After this, the background spectrum was collected. During the test, relative IR absorbance spectra were registered every 5 min in 4000–650 cm^−1^ wavenumber region with resolution 4 cm^−1^ and accumulation of 4 scans (0.7 min for a spectrum collection). From the beginning of the measurements, the samples were exposed to purified air flux for 30 min more to verify that baseline was zero. Next, the flux (100 mL/min) was switched to the test gas; it was purged for 1 h at a constant temperature in the range of 25–300 °C. The test gas was SO_2_ (10 ppm, 20 ppm, 40 ppm) in purified air. The source of test gas was certified gas mixture of SO_2_ (96 ± 5 ppm):N_2_ (Linde-Gas, Moscow, Russia).

To perform DC-resistance measurements the sensor samples were prepared. Powders were ground with a binder (terpeniol). The obtained paste was drop-deposited onto alumina microhotplates provided with vapor-deposited rectangular-shaped Pt contacts (0.3 mm × 0.2 mm) separated by 0.2 mm gap and with embedded Pt-heaters. The sensing layer (5–7 μm thick) covered area of 1.0 mm × 0.5 mm. As-prepared sensors were placed in a flow chamber of a resistance measuring device. Prior to measurements, the sensors were annealed at 300 °C for 14 h under purified air flux (100 mL/min) to remove the binder and/or to clean the surface from adsorbed humidity. Resistance measurements were performed *in situ* during the sensors exposure to a gas flow (100 mL/min) at a constant temperature in the range 100–450 °C. Temperature dependence of resistance was measured under purified air. The resistance dependence on oxygen concentration was measured using gas mixtures of purified air with analytical grade Ar (0-2-4-8-14-20 vol.% O_2_). In sensing tests, the flow was purged through the chamber in a pulsed regime: background gas—test gas—background gas, *etc*., the exposure time was 15 min, recovery time −45 min. Purified air was used as the background gas. The test gases were: CO (5–50 ppm), NH_3_ (2–20 ppm), NO (1–10 ppm), H_2_S (0.2–2 ppm), NO_2_ (0.2–2 ppm), H_2_ (100–1000 ppm), SO_2_ (1–10 ppm), EtOH (20–200 ppm), MeOH (20–200 ppm) and CO_2_ (1%). The analyte sources were certified gas mixtures: CO (517 ± 12 ppm):N_2_, NH_3_ (209 ± 8 ppm):air, NO (98 ± 8 ppm), H_2_S (51 ± 2 ppm):N_2_, NO_2_ (21.4 ± 0.9 ppm):N_2_, H_2_ (1.0 ± 0.1%):N_2_, SO_2_ (96 ± 4 ppm):N_2_, CO_2_ (10.4 ± 0.5%):air; liquid EtOH and MeOH (Sigma-Aldrich, St. Louis, MO, USA). Test gases were prepared by the analytes dilution in purified air. The sensor signal *S = R_air_/R_gas_* was defined as a ratio of resistance under background gas (*R_air_*) to that under test gas (*R_gas_*).

## 4. Conclusions

Cubic perovskite-type barium stannate was synthesized via aqueous coprecipitation with microwave-assisted hydrothermal treatment. Its particle size of 18–23 nm and BET surface area of 5–10 m^2^/g were unaffected by annealing temperature in the range 275–700 °C. Raising the annealing temperature to 1000 °C resulted in twofold increase of BaSnO_3_ crystallite size and decrease of BET surface area along with the elimination of carbonate impurity. Nanocrystalline tin dioxide was synthesized for comparison, its particle size (from 3–6 nm to 26–33 nm) and BET surface area (from <5 to 95–100 m^2^/g) being strongly dependent on annealing temperature in the range 300–1000 °C. Barium stannate showed *n*-type semiconductor behavior with the activation energy of 0.14–0.15 eV at 150–300 °C supposedly due to electron hopping mechanism of conductance. At higher temperature (350–450 °C), the activation energy decreased with the increase of BaSnO_3_ crystallinity, its average value being comparable to the ionization energy of oxygen vacancies. Comparative tests of sensor responses of BaSnO_3_ and SnO_2_ to ppm-level traces of various target gases in air were performed. An increased SO_2_ sensitivity of barium stannate was observed, despite tin dioxide having one order of magnitude larger BET area and smaller particle size. The reason for it was revealed from *in situ* DRIFT study of the materials interaction with SO_2_. Although adsorption at room temperature proceeded similarly on BaSnO_3_ and SnO_2_, at raised temperature the interaction routes were distinct. Unlike tin dioxide on the surface of which mostly molecular adsorption occurred, on the surface of BaSnO_3_ the oxidation of SO_2_ was the predominant process. Barium-promoted formation of surface sulfate species was recognized as the factor favoring target gas molecules oxidation that is responsible for increased SO_2_ sensitivity of barium stannate.

## References

[1] Eranna G., Joshi B.C., Runthala D.P., Gupta R.P. (2004). Oxide materials for development of integrated gas sensors—A comprehensive review. Crit. Rev. Solid State Mater. Sci..

[2] Morrison S.R. (1977). The Chemical Physics of Surfaces.

[3] Rumyantseva M.N., Gaskov A.M. (2008). Chemical modification of nanocrystalline metal oxides: Effect of the real structure and surface chemistry on the sensor properties. Russ. Chem. Bull..

[4] Hosono H. (2006). Ionic amorphous oxide semiconductors: Material design, carrier transport, and device application. J. Non Cryst. Solids.

[5] Kamiya T., Hosono H. (2010). Material characteristics and applications of transparent amorphous oxide semiconductors. NPG Asia Mater..

[6] Hosono H. (2007). Recent progress in transparent oxide semiconductors: Materials and device application. Thin Solid Films.

[7] Lampe U., Gerblinger J., Meixner H. (1995). Carbon-monoxide sensors based on thin films of BaSnO_3_. Sens. Actuators B.

[8] Lampe U., Gerblinger J., Meixner H. (1995). Nitrogen oxide sensors based on thin films of BaSnO_3_. Sens. Actuators B.

[9] Gopal Reddy C.V., Manorama S.V., Rao V.J. (2001). Preparation and characterization of barium stannate: Application as a liquefied petroleum gas sensor. J. Mater. Sci. Mater. Electron..

[10] Cerda J., Arbiol J., Dezanneau G., Dıaz R., Morante J.R. (2002). Perovskite-type BaSnO_3_ powders for high temperature gas sensor applications. Sens. Actuators B.

[11] Yasukawa M., Kono T., Ueda K., Yanagi H., Hosono H. (2010). High-temperature thermoelectric properties of La-doped BaSnO_3_ ceramics. Mater. Sci. Eng. B.

[12] Upadhyay S., Parkash O., Kumar D. (2001). Solubility of lanthanum, nickel and chromium in barium stannate. Mater. Lett..

[13] Lu W., Jiang S., Zhou D., Gong S. (2000). Structural and electrical properties of Ba(Sn,Sb)O_3_ electroceramics materials. Sens. Actuators B.

[14] Larramona G., Gutierrez C., Pereira I., Rosa Nunes M., da Costa F.M.A. (1989). Characterization of the mixed perovskite BaSn_1-x_Sb_x_O_3_ by electrolyte electroreflectance, diffuse reflectance, and X-Ray photoelectron spectroscopy. J. Chem. Soc., Faraday Trans. 1.

[15] Cava R.J., Gammel P., Batlogg B., Krajewski J.J., Peck W.F., Rupp L.W., Felder R., van Dover R.B. (1990). Nonsuperconducting BaSn_1-x_Sb_x_O_3_: The 5s-orbital analog of BaPb_1-x_Bi_x_O_3_. Phys. Rev. B.

[16] Upadhyay S., Parkash O., Kumar D. (2007). Synthesis, structure and electrical behaviour of nickel-doped barium stannate. J. Alloys Compd..

[17] Doroftei C., Popa P.D., Iacomi F. (2012). Study of the influence of nickel ions substitutes in barium stannates used as humidity resistive sensors. Sens. Actuators A.

[18] Mizoguchi H., Woodward P.M., Park C.H., Keszler D.A. (2004). Strong near-infrared luminescence in BaSnO_3_. J. Am. Chem. Soc..

[19] Scanlon D.O. (2013). Defect engineering of BaSnO_3_ for high-performance transparent conducting oxide applications. Phys. Rev. B.

[20] Mizoguchi H., Eng H.W., Woodward P.M. (2004). Probing the Electronic Structures of Ternary Perovskite and Pyrochlore Oxides Containing Sn^4+^ or Sb^5+^. Inorg. Chem..

[21] Moreira E., Henriques J.M., Azevedo D.L., Caetano E.W.S., Freire V.N., Fulco U.L., Albuquerque E.L. (2012). Structural and optoelectronic properties, and infrared spectrum of cubic BaSnO_3_ from first principles calculations. J. Appl. Phys..

[22] Kuferstein R., Yakuphanoglu F. (2010). Semiconducting properties of Ge-doped BaSnO_3_ ceramic. J. Alloys Compd..

[23] Upadhyay S., Parkash O., Kumar D. (1997). Preparation and characterization of barium stannate BaSnO_3_. J. Mater. Sci. Lett..

[24] Bajpai P.K., Ratre K., Pastor M., Sinha T.P. (2003). Preparation, characterization and dielectric behaviour of some yttrium doped strontium stannates. Bull. Mater. Sci..

[25] Kutty T.R.N., Vivekanadan R. (1987). BaSnO_3_ fine powders from hydrothermal preparations. Mater. Res. Bull..

[26] Lu W., Schmidt H. (2007). Preparation and characterization of BaSnO_3_ powders by hydrothermal synthesis from tin oxide hydrate gel. J. Mater. Sci..

[27] Guo F., Li G., Yang N., Wang W., Zhang W. (2012). Preparation and photophysical properties of rhombic dodecahedral perovskite-type BaSnO_3_. Appl. Phys. A.

[28] Udawatte C.P., Yoshimura M. (2001). Preparation of well-crystallized BaSnO_3_ powders under hydrothermal conditions. Mater. Lett..

[29] Lu W., Schmidt H. (2008). Lyothermal synthesis of nanocrystalline BaSnO_3_ powders. Ceram. Int..

[30] Cerda J., Arbiol J., Diaz R., Dezanneau G., Morante J.R. (2002). Synthesis of perovskite-type BaSnO_3_ particles obtained by a new simple wet chemical route based on a sol–gel process. Mater. Lett..

[31] Tao S., Gao F., Liu X., Sorensen O.T. (2000). Ethanol-sensing characteristics of barium stannate prepared by chemical precipitation. Sens. Actuators B.

[32] Udawatte C.P., Kakihana M., Yoshimura M. (1998). Preparation of pure perovskite-type BaSnO_3_ powders by the polymerized complex method at reduced temperature. Solid State Ionics.

[33] Upadhyay S., Kavitha P. (2007). Lanthanum doped barium stannate for humidity sensor. Mater. Lett..

[34] Ostrick B., Fleischer M., Lampe U., Meixner H. (1997). Preparation of stoichiometric barium stannate thin films: Hall measurements and gas sensitivities. Sens. Actuators B.

[35] Kumari U.S., Suresh P., Prasada Rao A.V. (2013). Solid-state metathetic synthesis of phase pure BaSnO_3_ and BaZrO_3_. Int. Res. J. Pure Appl. Chem..

[36] Barsan N., Weimar U. (2001). Conduction model of metal oxide gas sensors. J. Electroceram..

[37] Rumyantseva M.N., Makeeva E.A., Badalyan S.M., Zhukova A.A., Gaskov A.M. (2009). Nanocrystalline SnO_2_ and In_2_O_3_ as materials for gas sensors: The relationship between microstructure and oxygen chemisorption. Thin Solid Films.

[38] Kocemba I., Wrobel-Jedrzejewska M., Szychowska A., Rynkowski J., Glowka M. (2007). The properties of barium stannate and aluminum oxide-based gas sensor. The role of Al_2_O_3_ in this system. Sens. Actuators B.

[39] Kilic C., Zunger A. (2002). Origins of coexistence of conductivity and transparency in SnO_2_. J. Phys. Rev. Lett..

[40] Gopal Reddy C.V., Manorama S.V., Rao V.J., Lobo A., Kulkarni S.K. (1999). Noble metal additive modulation of gas sensitivity of BaSnO_3_, explained by a work function based model. Thin Solid Films.

[41] Manorama S.V., Gopal Reddy C.V., Rao V.J. (2001). X-ray photoelectron spectroscopic studies of noble metal-incorporated BaSnO_3_ based gas sensors. Appl. Surf. Sci..

[42] Islam M.N., Hakim M.O. (1986). Electron affinity and work function of polycrystalline SnO_2_ thin film. J. Mater. Sci. Lett..

[43] Kanan S.M., El-Kadri O.M., Abu-Yousef I.A., Kanan M.C. (2009). Semiconducting metal oxide based sensors for selective gas pollutant detection. Sensors.

[44] Karge H.G., Dalla Lana I.G. (1984). Infrared studies of SO_2_ adsorption on a Claus catalyst by selective poisoning of sites. J. Phys. Chem..

[45] Nakamoto K. (2009). Infrared and Raman Spectra of Inorganic and Coordination Compounds. Part B: Applications in Coordination, Organometallic and Bioinorganic Chemistry.

[46] Marcu I.C., Sandulescu I. (2004). Study of sulfur dioxide adsorption on Y zeolite. J. Serb. Chem. Soc..

[47] Periasamy A., Muruganand S., Palaniswamy M. (2009). Vibrational studies of Na_2_SO_4_, K_2_SO_4_, NaHSO_4_ and KHSO_4_ crystals. RASAYAN J. Chem..

[48] Kalbus G.E., Lieu V.T., Kalbus L.H. (2006). Infrared examination of the transformation of barium sulfate into barium carbonate. J. Chem. Educ..

